# Meta-analysis of the therapeutic effects of traumatic BPPV and idiopathic BPPV

**DOI:** 10.3389/fneur.2026.1834081

**Published:** 2026-06-09

**Authors:** Yifei Fu, Zhibin Zhao

**Affiliations:** Department of Otolaryngology Head and Neck Surgery, Hainan General Hospital, Hainan Affiliated Hospital of Hainan Medical University, Xiuying, Haikou, China

**Keywords:** benign paroxysmal positional vertigo, idiopathic, meta-analysis, repositioning, traumatic

## Abstract

**Objective:**

To systematically evaluate differences in treatment efficacy and prognosis between traumatic benign paroxysmal positional vertigo (t-BPPV) and idiopathic BPPV (i-BPPV), and to provide evidence-based guidance for clinical practice.

**Methods:**

We systematically searched 8 databases (PubMed, EMBASE, Cochrane Library, Web of Science, CBMdisc, Wanfang, CNKI, and VIP) for cohort studies comparing t-BPPV and i-BPPV, with the search period extending until September 2023. Two independent reviewers screened literature, extracted data, and assessed methodological quality using the Newcastle-Ottawa Scale (NOS). Meta-analyses were performed using RevMan 5.3 and Stata 12.0. Risk ratios (RR) with 95% confidence intervals (CI) were calculated for dichotomous outcomes. Heterogeneity was evaluated via *p*-values and I^2^statistics, with fixed-effects models applied for I^2^ ≤ 50% and random-effects models otherwise.

**Results:**

Seven high-quality cohort studies (NOS ≥ 6) involving 4,074 patients were included. Compared with i-BPPV, t-BPPV demonstrated significantly poorer outcomes across all metrics: higher recurrence rate (RR = 3.39, 95%CI 3.07–3.74, *p* < 0.00001), greater repositioning difficulty (RR = 3.05, 95%CI 2.74–3.40, *p* < 0.00001), lower treatment efficacy (RR = 0.87, 95%CI 0.84–0.90, *p* < 0.00001), higher proportion of multi-semicircular canal involvement (RR = 2.91, 95%CI 1.55–5.47, *p* = 0.0009), and elevated bilateral BPPV incidence (RR = 3.37, 95%CI 1.86–6.10, *p* < 0.00001). Egger’s test revealed no significant publication bias for any outcome (*p* > 0.05).

**Conclusion:**

t-BPPV exhibits inferior repositioning efficacy, higher risks of residual symptoms and recurrence, and increased rates of multi-semicircular canal and bilateral involvement compared with i-BPPV. Clinicians should adopt tailored management strategies for t-BPPV, emphasizing long-term follow-up and personalized interventions.

## 1 Introduction

Benign paroxysmal positional vertigo (BPPV) is the most prevalent peripheral vestibular disorder, accounting for approximately one-third of all vertigo-related outpatient visits (1). It is characterized by transient, intense vertigo and torsional or horizontal nystagmus induced by specific changes in head position relative to gravity (2). While BPPV is typically a self-limiting condition with the potential for spontaneous resolution, it significantly impairs patients’ quality of life. The first-line treatment involves canalith repositioning procedures (CRP), such as the Epley maneuver, which utilize gravity to relocate displaced otoconia from the semicircular canals back to the utricle (3).

Based on etiology, BPPV is broadly categorized into idiopathic (i-BPPV) and secondary forms. Approximately 50 to 70% of cases are classified as i-BPPV, where no definitive cause is identified (4, 5). The remaining cases are secondary to various pathologies, including otologic diseases (e.g., sudden sensorineural hearing loss, vestibular neuritis), systemic conditions (e.g., migraines, diabetes), or physical trauma (6–8). Specifically, BPPV secondary to head trauma, neurosurgery, or maxillofacial surgery is defined as traumatic BPPV (t-BPPV), representing roughly 8.5 to 18% of the total BPPV population (9, 10).

Although i-BPPV predominantly affects the elderly and females, and usually involves a single canal with a favorable response to repositioning, t-BPPV presents distinct clinical features. Previous studies suggest that t-BPPV occurs equally among sexes, peaks in a younger demographic, and is associated with a higher incidence of bilateral involvement, multi-canal involvement, and increased recurrence rates (11). However, these conclusions remain controversial, as some researchers argue that there are no significant differences in recurrence or treatment difficulty between t-BPPV and i-BPPV (12, 13). Furthermore, the diagnostic criteria for t-BPPV, particularly the time interval between trauma and symptom onset, lack standardization across studies.

Given these inconsistencies and the absence of high-level evidence guiding clinical management, there is a critical need to synthesize existing data. To address this gap, we conducted a meta-analysis exclusively including cohort studies to compare the treatment efficacy and prognosis between t-BPPV and i-BPPV. By pooling quantitative data, this study aims to clarify whether t-BPPV constitutes a more refractory subtype of vertigo, thereby providing robust evidence for optimizing therapeutic strategies.

## 2 Materials and methods

### 2.1 Data sources

We conducted a comprehensive search of eight databases: PubMed, EMBASE, Cochrane Library, Web of Science, CBMdisc, Wanfang Database, CNKI, and VIP Database. The search strategy combined Medical Subject Headings (MeSH) and free-text terms. The search period spanned from the inception of each database to September 2023. Grey literature was supplemented by manually tracing references and contacting experts in the field. The search terms included: (“Benign Paroxysmal Positional Vertigo” OR “BPPV”) AND (“Trauma” OR “Traumatic Brain Injury” OR “TBI”) AND (“Idiopathic” OR “Primary”).

### 2.2 Inclusion criteria

(1) Study type: cohort study (retrospective or prospective), language types were Chinese and English. (2) Research object: the comparative study of t-BPPV and i-BPPV includes at least one t-BPPV group and one i-BPPV group. (3) The diagnosis of BPPV is clear, that is, ① the brief and repeated vertigo attacks caused by the change of head position relative to the gravity vector; ② Characteristic nystagmus and vertigo occurred in position test. Recurrence was initially defined as BPPV recurred with positional vertigo consistent with the initial attack after successful otolith reduction, or typical nystagmus after positional test; (4) Observation index: it can provide the relevant specific data of the times of reduction treatment and (or) recurrence rate of patients in t-BPPV group and i-BPPV group. (5) Outcome measures: recurrence rate, recurrence repositioning difficulty, proportion of the affected semicircular canals, proportion of bilateral BPPV, and treatment effect.

### 2.3 Definitions of outcome measures

To ensure analytical rigor, outcomes were predefined as follows:

(1) Recurrence Rate: Occurrence of vertigo and positional nystagmus after a documented symptom-free interval post-treatment. (2) Repositioning Difficulty: Operationalized as requiring ≥2 maneuvers for complete resolution. (3) Treatment Effect: Immediate resolution of symptoms and nystagmus after the first maneuver. (4) Multi-Semicircular Canal Involvement: Concurrent BPPV in two or more canals of the same ear. (6) Bilateral BPPV: Simultaneous BPPV lesions in both ears.

### 2.4 Exclusion criteria

① The efficacy judgment index is not clear or standard; ② Review, commentary and guide literature; ③ Self controlled study; ④ The baseline of the study subjects was unclear or the general information was not comparable.

### 2.5 Literature screening and data extraction

Evaluators screened the literature according to the inclusion criteria strictly by reading the title, abstract and full text. The extracted data included research interventions, methods used and possible bias, journal name, first author, publication time, sample inclusion criteria, sample size, baseline characteristics of research subjects, outcome indicators, etc. EndNote X9 is used to screen for and remove duplicate entries, retaining only unique citations. Two evaluators independently performed data statistics, and the lack of data was obtained by contacting the author.

### 2.6 Literature quality evaluation

In this paper, the Newcastle Ottawa scale (NOS) was used to evaluate the methodological quality of the included literature. NOS belongs to the observational research evaluation scale, and its indicators include: study object selection (including 4 items, a total of 4 points), comparability between groups (including 1 item, a maximum of 2 points), exposure factor measurement (including 3 items, a total of 3 points). The maximum score of quality evaluation is 9 points, and ≥ 6 points can be considered as high quality literature.

### 2.7 Publication bias

Egger’s method was used to test the symmetry of the funnel plot. If *p* < 0.05, it suggested that the funnel plot was asymmetric, indicating that the outcome index had publication bias; otherwise, there was no publication bias. Egger’s test calculation results were calculated and generated by Stata12.0.

### 2.8 Statistical analysis

Revman 5.2 software was used for data analysis. The relative risk (*RR*) was used as the effect size for dichotomous data, and the standardized mean difference (*SMD*) was used as the effect size for continuous data. Both gave 95% confidence interval (*CI*). The *p* value of the heterogeneity test results displayed in the forest plot is used to judge whether the included studies have heterogeneity, and the heterogeneity is judged according to *I*^2^. If the heterogeneity was not statistically significant (*p* ≥ 0.1, *I* ^2^ ≤ 50%), the fixed effect model was used to combine the effect sizes; If heterogeneity is statistically significant (*p* < 0.1, *I*^2^ > 50%), the source of heterogeneity is analyzed, and sensitivity analysis is used. If the reason for heterogeneity cannot be explained, and these studies have clinical homogeneity, the random effect model is used to combine the effect size.

## 3 Results

### 3.1 Search results and basic characteristics of included studies

**Figure 1:**
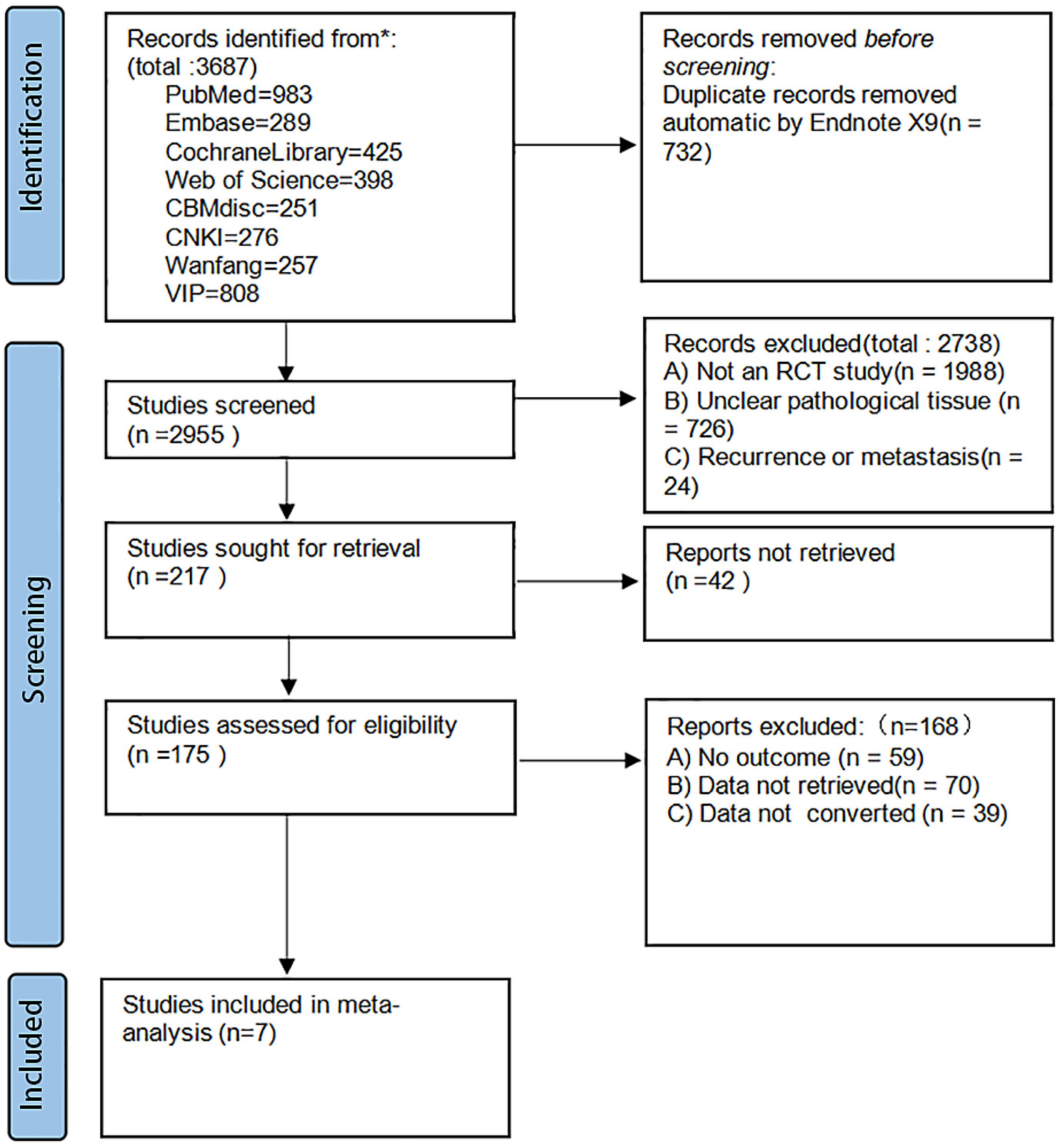
Flow chart of literature screening.

Three thousand six hundred seventy-five articles were obtained through preliminary screening, and 12 articles were obtained through tracking references. ① Excluding the literature repeatedly included in different databases (732 articles); ② Read titles and abstracts and eliminate Literature (2738); ③ Read the full text, screen according to the inclusion criteria, and exclude case reports, clinical symptom analysis, reviews, and abstracts of meeting contents for which the full text cannot be obtained (42); ④ Seven literatures were finally included, including 4,074 patients. The literature screening process is shown in Figure 1, the basic information of the included literature is shown in Table 1, and the basic information of patients is shown in Table 2.

**Table 1:** Basic information of included literatures.

**First author**	**Year**	**Country**	**grouping**	**Number of cases**	**Age**	**Gender (male)**	**Interval between trauma and BPPV**	**Type of trauma**	**Follow up time**	**Outcome measures**	**NOS** **score**
Ahn (9)	2011	Korea	T-BPPV	32	52.9 ± 11.2	18	≤3 days	Traffic accident 20; Fall 8; Head injury 4	≥ 6 months Traumatic: 32.4 ± 9.3 months Idiopathic: 35.2 ± 9.9 months	① ② ③ ④ ⑤	7
			I-BPPV	112	55.3 ± 15.9	44					
Dimitrios (17)	2017	Greece	T-BPPV	33	43.1 ± 7.7	17	≤ 2 weeks	Mild traumatic brain damage Injury 33	12 months	① ② ③ ④ ⑤	7
			I-BPPV	320	55.1 ± 14.4	125					
Gordon (14)	2005	Israel	T-BPPV	21	56.3 ± 15.6	10	≤3 days	Traffic accident 12; Fall 4; Head injury 1; Surgery 3; By speedboat 1	June to December Average 21.7 ± 9.7 months	① ② ④	8
			I-BPPV	42	61.1 ± 22.3	10					
Guo (18)	2010	China	T-BPPV	23	52 ± 17	12	≤3 days	Traffic accident 11; head Knock injury 6; Fall 3; Surgery 3	≥2 years	① ② ④ ⑤	8
			I-BPPV	163	56 ± 13	49					
Liu (15)	2012	China	T-BPPV	40	/	/	≤March	Traumatic brain injury 40	≥1 year	① ② ③ ④	7
			I-BPPV	46	/	/					
Pisani (16)	2015	Italy	T-BPPV	716	56.8 ± 16.6	311	≤2 weeks	Road collision 422; Falls 199; Strenuous exercise 26; Surgery 21; Massage and gymnastics 10; Recumbent Bed 33	≥1 year	① ② ③ ⑤	8
			I-BPPV	2,344	60.8 ± 14.4	837					
Wang (19)	2021	China	T-BPPV	118	44 ± 11.2	62	≤ 3 days	31 cases were injured by falling; 69 cases were injured in traffic accident; 18 cases of head injury; There were 80 cases ipsilateral to head trauma and 38 cases contralateral to head trauma.	≥ 1 year	① ⑤	7
			I-BPPV	188	54 ± 12.2	70					

Outcome measures ① recurrence rate, ② recurrence repositioning difficulty, ③ majority regulatory ratio, ④ bilateral BPPV ratio, ⑤ treatment effect.

**Table 2:** Basic information of patients included in the literature.

First author	Grouping	Number of cases (t/i)	BPPV involvement semicircular canal Psc/lsc/asc	Most regulation BPPV	Bilateral BPPV	First repositioning successful cases number	≥ 2 times number of repositioning cases	No recurrence number of cases	Recrudescence number of cases
Ahn (9)	T-BPPV	32	24/11/0	0	4	18	11	27	5
	I-BPPV	112	72/44/0	0	0	94	11	91	21
Dimitrios (17)	T-BPPV	33	13/8/4	8	5	6	27	16	8
	I-BPPV	320	239/38/18	24	26	242	78	279	41
Gordon (14)	T-BPPV	21	21/0/0	0	4	7	14	9	12
	I-BPPV	42	43/0/0	0	0	36	6	34	8
Guo (18)	T-BPPV	23	23/0/0	-The	4	8	15	10	13
	I-BPPV	163	163/0/0	-The	11	139	24	132	31
Liu (15)	T-BPPV	40	-The	-The	-The	14	26	13	27
	I-BPPV	46	-The	-The	-The	39	7	42	4
Pisani (16)	T-BPPV	716	544/121/0	51	-The	338	313	254	397
	I-BPPV	2,344	1805/452/0	87	-The	1,521	773	1881	413
Wang (19)	T-BPPV	118	-The	-The	-The	-The	-The	-The	-The
	I-BPPV	188	-The	-The	-The	-The	-The	-The	-The

### 3.2 Quality evaluation

According to the recommendation of the non randomized research methodology group of the Cochrane Collaboration, we used NOS to evaluate the quality of all the included literatures. The seven included literatures were high-quality studies (NOS score of 7–8 stars), see Table 1. The main deduction item of the 7 literatures is the outcome indicator that has been observed at the beginning of the study, because all studies are retrospective studies. In addition, two literatures (14, 15) did not clearly describe the specific situation of exposure factor trauma.

### 3.3 Meta-analysis results

#### 3.3.1 Recurrence rate

**Figure 2:**
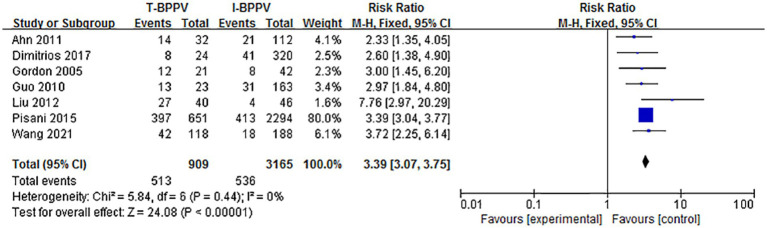
Forest plot of meta-analysis of recurrence rate in t-BPPV and i-BPPV treatment.

Seven literatures (9, 14–19) reported the number of non relapse and relapse in t-BPPV and i-BPPV groups, and the heterogeneity analysis showed that *p =* 0.44, *I*^2^ = 0%. There was no obvious heterogeneity among the studies. The fixed effect model was used for meta-analysis, and the results were statistically significant [*RR* = 3.39, 95% *CI* (3.07–3.74), *p* < 0.00001], indicating that the recurrence rate of t-BPPV group was significantly different from that of i-BPPV treatment group, and the recurrence rate of t-BPPV group was higher than that of i-BPPV group, as shown in Figure 2.

#### 3.3.2 Recurrence repositioning difficulty

**Figure 3:**
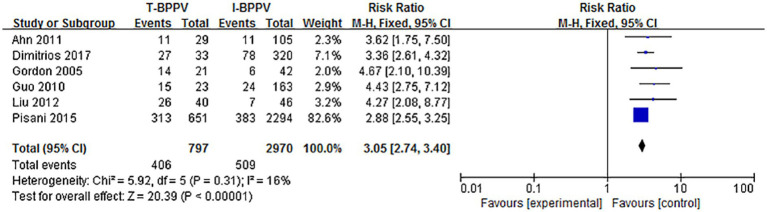
Forest diagram of meta-analysis of recurrence repositioning difficulty in t-BPPV group and i-BPPV treatment group.

Six literatures (9, 14–18) reported the number of repositioning in t-BPPV group and i-BPPV group, which indicates the difficulty of repositioning, and ≥ 2 indicates the difficulty of repositioning. Meta-analysis showed that heterogeneity analysis showed *p* = 0.31, *I*^2^ = 16%. There was no obvious heterogeneity among the studies. The fixed effect model was used for meta-analysis, and the results showed that there was statistical significance between the two groups [*RR* = 3.05, 95%*CI* (2.74 ~ 3.40), *p* < 0.00001]. The results showed that there was a significant difference in the difficulty of recurrence between the t-BPPV group and the i-BPPV treatment group. Compared with the i-BPPV group, the t-BPPV group was more prone to recurrence, as shown in Figure 3.

#### 3.3.3 Treatment effect

**Figure 4:**
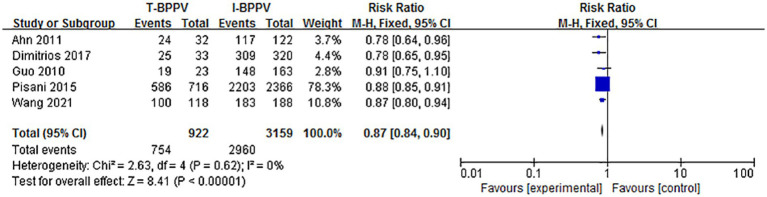
Forest diagram of meta-analysis of treatment effect of t-BPPV and i-BPPV treatment groups.

Five literatures (9, 16–19) reported the therapeutic effects of t-BPPV group and i-BPPV group. Heterogeneity analysis showed that *p =* 0.62, *I*^2^ = 0%. There was no heterogeneity among the studies. The fixed effect model was used for meta-analysis, and the results showed that the two groups had statistical significance [*RR* = 0.87, 95%*CI* (0.84 ~ 0.90), *p* < 0.00001], indicating that there was a significant difference in the treatment effect between t-BPPV and i-BPPV, and the treatment effect of t-BPPV group was lower than that of i-BPPV group, as shown in Figure 4.

#### 3.3.4 MSC-BPPV ratio

**Figure 5:**
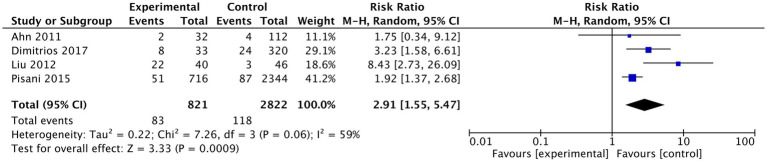
Forest plot of meta-analysis of the proportion of t-BPPV and i-BPPV multi semicircular canals.

Four literatures (9, 15–17) reported the proportion of t-BPPV and i-BPPV groups, MSC-BPPV and all BPPV. Heterogeneity analysis showed that *p =* 0.06, *I*^2^ = 59%. There was heterogeneity among the studies. The random effects model was used for meta-analysis, and the results showed that the two groups had statistical significance [*RR* = 2.91, 95% *CI* (1.55–5.47). *p =* 0.0009], indicating that there was a significant difference in the proportion of multiple semicircular canals between the t-BPPV group and the i-BPPV group. The proportion of multiple semicircular canals in the t-BPPV group was higher than that in the i-BPPV group, as shown in Figure 5.

#### 3.3.5 Proportion of bilateral BPPV

**Figure 6:**
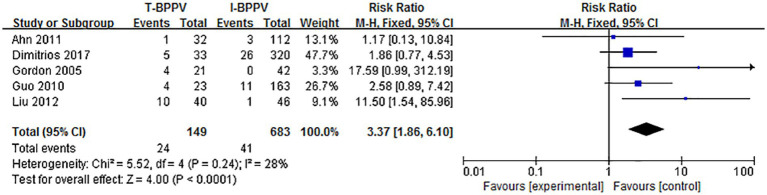
Forest plot of meta-analysis on the difference of bilateral BPPV between t-BPPV and i-BPPV treatment.

Five literatures (9, 14, 15, 17, 18) reported the difference in the proportion of bilateral BPPV to total BPPV between t-BPPV and i-BPPV. Heterogeneity analysis showed that *p =* 0.24, *I*^2^ = 28%. There was no obvious heterogeneity among the studies, and the fixed effect model was used for meta-analysis. The results showed that there was a statistically significant difference between the two groups [*RR* = 3.37, 95%*CI*(1.86 ~ 6.10), *p* < 0.00001], indicating that there was a significant difference in the proportion of bilateral BPPV between t-BPPV group and i-BPPV group, and the proportion of bilateral BPPV in t-BPPV group was higher than that in i-BPPV group, as shown in Figure 6.

### 3.4 Publication bias

The number of outcome indicators affected by this study is small, and the significance of funnel plot asymmetry test is not significant. Therefore, Egger’s method was used to test the funnel plot symmetry of the recurrence rate, the degree of recurrence repositioning difficulty, the proportion of the affected semicircular canals regulation, the proportion of bilateral BPPV, and the treatment effect; Egger’s method test calculation results *p* value were 0.236, 0.451, 0.973, 0.236, 0.594, *p-*values were > 0.05, suggesting that the outcome index does not have obvious publication bias, see Table 3.

**Table 3:** Egger’s method test calculation results.

**Std_Eff**	**Coef.**	**Std.**	**Err.**	***t***	**P > |*t*|**	**[95% Conf. Interval]**
Slope	2.763719	0.1162177	23.78	0.000	2.441047	3.086391
Bias	3.662962	4.392547	0.83	0.451	−8.532704	15.85863

**Std_Eff**	**Coef.**	**Std.**	**Err.**	***t***	**P > |*t*|**	**[95% Conf. Interval]**
Slope	2.763719	0.1162177	23.78	0.000	2.441047	3.086391
Bias	3.662962	4.392547	0.83	0.451	−8.532704	15.85863

**Std_Eff**	**Coef.**	**Std.**	**Err.**	***t***	**P > |*t*|**	**[95% Conf. Interval]**
Slope	2.082826	0.3103804	6.71	0.021	0.7473665	3.418285
Bias	0.750753	1.955359	0.04	0.973	−8.338155	8.488306

**Std_Eff**	**Coef.**	**Std.**	**Err.**	***t***	**P > |*t*|**	**[95% Conf. Interval]**
Slope	1.686167	0.1006292	16.76	0.004	1.253194	2.119139
Bias	−1.463075	0.87355514	−1.67	0.236	−5.221663	2.295513

**Std_Eff**	**Coef.**	**Std.**	**Err.**	***t***	**P > |*t*|**	**[95% Conf. Interval]**
Slope	4.973862	0.8082756	6.15	0.009	2.410568	7.546156
Bias	2.238194	3.767198	0.59	0.594	−9.750711	14.2271

### 3.5 Sensitivity analysis

**Figure 7:**
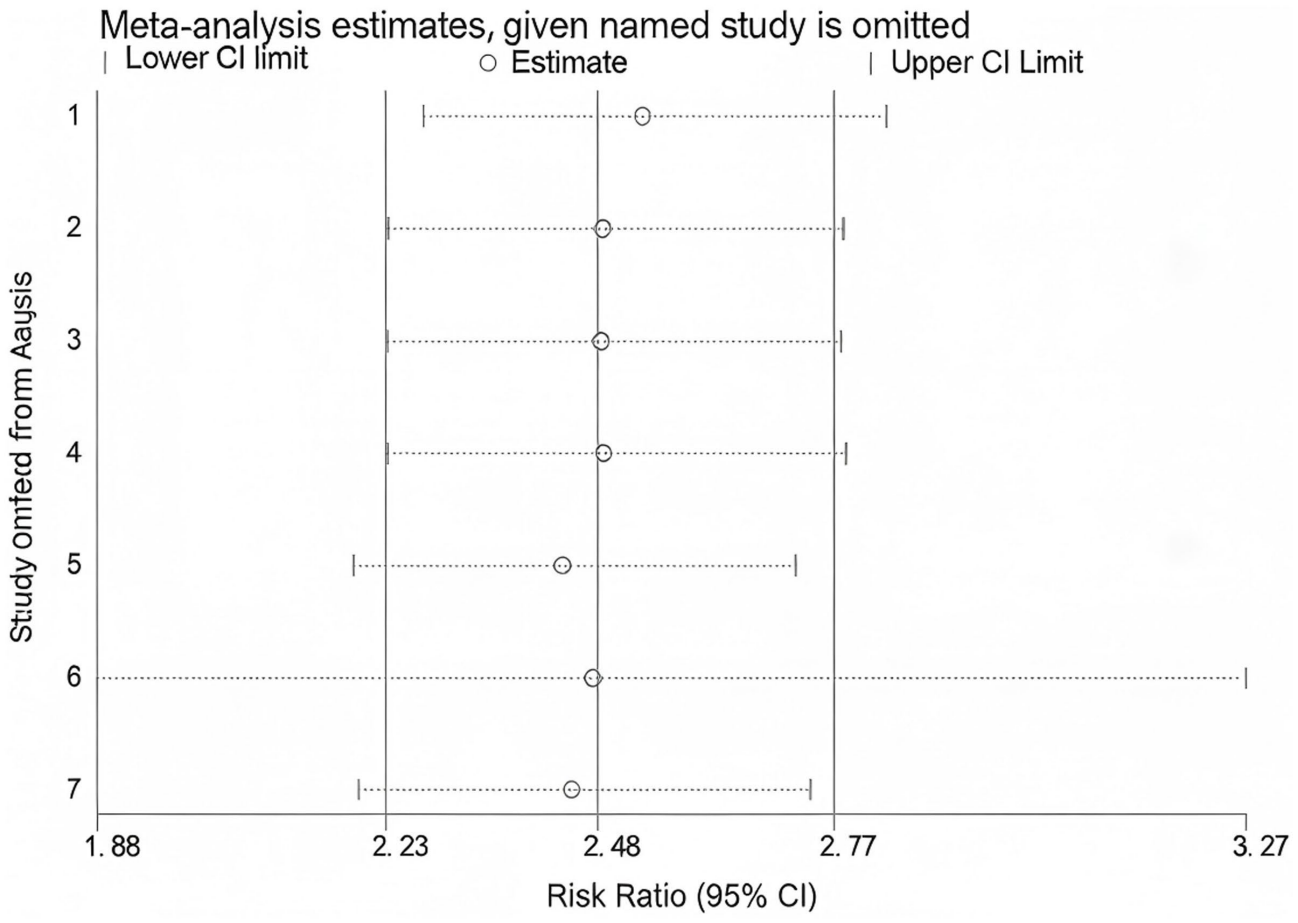
Sensitivity analysis chart.

Sensitivity analysis was conducted on the recurrence rate with the largest number of main outcome indicators. It can be seen that the research values of seven studies fell within the 95% confidence interval, indicating that the included literature was stable and could be analyzed in the next step. The difference was still statistically significant and the structural change of forest plot was not obvious, indicating that the meta-analysis results were reliable, see Figure 7.

## 4 Discussion

Vertigo is a common disease and also a difficult disease, with a lifetime prevalence of 20–30% (20), and BPPV ranks first in the disease group of vertigo, with a lifetime prevalence of 10% (21). Frequent attacks of vertigo have plagued patients for a long time, and also brought challenges to clinicians’ diagnosis and treatment. Trauma is a common cause of secondary BPPV, but the diagnostic criteria and pathogenesis of traumatic BPPV are still controversial. Some scholars believe that traumatic BPPV is caused by the strong acceleration stimulation of the otolith membrane of the utricle after head trauma, which causes the otolith to fall off and enter the semicircular canal (1, 22), and can cause one or both posterior semicircular canals or multiple canaliculi. They propose to include those with a clear history of trauma within 3 days before the onset of traumatic BPPV (23), Other scholars (24) believe that at the initial stage of head injury, the otolith membrane may only have structural changes without otolith falling off. After a certain period of time, the otolith falling off can cause vertigo. The onset within 30 days after injury is also classified as traumatic vertigo. In addition, due to the inconvenience of movement after head injury, bed rest or body position restriction is also one of the causes of BPPV.

Most of the patients who cannot find any etiology are classified as i-BPPV, and the other part of BPPV is secondary, in which trauma is the most common secondary factor (25). Usually, BPPV secondary to TBI, neck trauma, otolaryngology or oral and maxillofacial surgery or neurosurgery, physical exercise and other (26) various types of trauma is called t-BPPV. T-BPPV accounts for approximately 8.5–18% of all BPPV cases (27). Unfortunately, up to now, there is no accepted and precise definition of t-BPPV. Combined with previous studies, the definition of t-BPPV in this paper is that there is a clear record of trauma history, and the time interval between trauma and typical positional vertigo or BPPV diagnosis must be clearly stated, and the maximum time should not exceed 3 months. Aron (28) and others have made a systematic review of the clinical characteristics of t-BPPV and i-BPPV, especially the symptom relief rate. In view of the descriptive studies including case reports, case series reports, and cohort studies, no further meta-analysis was conducted. After Aron *et al*.’s study, there was another new cohort study on t-BPPV with a larger sample (29).

This meta-analysis, comprising 4,074 patients from seven cohort studies, provides robust evidence that t-BPPV exhibits a more refractory clinical profile than i-BPPV. The most striking finding was the three-fold increase in recurrence risk (RR = 3.39) and tripled repositioning difficulty (RR = 3.05) in the traumatic group. Unlike i-BPPV, which typically involves a single canal, t-BPPV frequently presents with multi-canal (RR = 2.91) and bilateral involvement (RR = 3.37). These substantial effect sizes suggest that trauma does not merely trigger BPPV but alters its underlying pathophysiology, likely through widespread otoconial dislocation affecting both ears and multiple canals simultaneously.

BPPV has a certain recurrence rate. This study also confirmed that the difference between t-BPPV and i-BPPV was statistically significant, suggesting that t-BPPV is easier to recur than i-BPPV, and patients need to be instructed to follow up regularly. This is consistent with our inclusion of literature reports. Most scholars believe that t-BPPV is more likely to recur than i-BPPV (30). The falling will last for at least 3 months. It is presumed that the ligament or protein connecting otolith particles with otolith membrane or other otolith particles may be damaged after trauma. In this way, the otolith particles gradually fall off for a long time, which may cause the recurrence of traumatic BPPV. Therefore, the secondary damage of otolith function after trauma may be an important reason for the frequent recurrence of t-BPPV.

As for the degree of difficulty and ease of reduction, this study confirmed that the difference between t-BPPV and i-BPPV was statistically significant, suggesting that the number of cycles of reduction required for the treatment of t-BPPV is more than that of i-BPPV, and the reduction is more difficult. In the study of Katsarkas *et al* (31), the single reduction success rate of i-BPPV patients was 86%, while that of t-BPPV patients was 33%, with statistically significant difference. This is also consistent with the previous systematic analysis by Aron *et al*. The study of Pisani *et al*. (18) has the largest sample size among the seven literatures we included, and it also includes the most types of trauma. In addition to the most common traffic accidents, falls, head injuries, it also includes strenuous exercise, massage, gymnastics, etc., which to a certain extent increases the degree of variation of the study. Statistics of the number of repositioning maneuvers. The reason why t-BPPV is difficult to repositioning or needs multi cycle repositioning may be that traumatic brain injury destroyed the normal anatomy of the semicircular canal membranous labyrinth, because magnetic resonance imaging of the inner ear showed the stenosis or filling defect of the semicircular canal. It is presumed that trauma may lead to a considerable number of otolith particles falling off, resulting in otolith blockage of the semicircular canal or otolith particles adhering to the cristae cap of the ampulla of the semicircular canal, increasing the difficulty of repositioning (32).

There is controversy about the rehabilitation effect of traumatic BPPV at home and abroad. Some scholars (33, 34) believe that compared with i-BPPV, t-BPPV has a poor short-term therapeutic effect; other scholars believe that there is no statistically significant difference in the short-term efficacy between the two (32). Our study found that the rehabilitation effect of t-BPPV group was significantly lower than that of i-BPPV group, and the difference between the two groups was statistically significant. The results are consistent with the report of Korres et al. (35). The reasons for the difficulty in rehabilitation treatment may be related to the large amount of otoliths shed due to trauma, extensive pathological changes in the vestibule (such as local slight bleeding or tissue degeneration and loosening), structural abnormalities, and the continuous or intermittent shedding of otoliths caused by these changes (36). Existing studies have shown that the residual symptoms of BPPV patients after reduction treatment may originate from a small amount of otolith residue after otolith reduction, otolith injury (37) and homologous semicircular canal injury (38). The high rate of residual symptoms of traumatic BPPV suggests that it may have relatively severe otolith lesions.

This meta-analysis also confirmed that the incidence of MSC-BPPV and bilateral BPPV was high. Liu *et al* (15) defined t-BPPV as the time interval between trauma and positional vertigo not exceeding 3 months, which was the longest time interval in all the included literature. Within 3 months, otolith particles may gradually fall off the damaged otolith membrane in batches and enter different semicircular canals to cause BPPV, which may be the reason for the high incidence of MSC-BPPV of 55%. For the meta-analysis of the incidence of bilateral BPPV, five literatures were included, and only the study of Pisani *et al* (18) did not count the incidence of bilateral BPPV. The heterogeneity test showed that the heterogeneity was small (I^2^ = 28%), indicating that the conclusion of high incidence of bilateral BPPV in t-BPPV has high reliability. In the study of Liu *et al* (15), the conclusion that the incidence of bilateral BPPV in t-BPPV patients is high has high credibility. There was no significant difference with i-BPPV patients. The high incidence of MSC-BPPV and bilateral BPPV in t-BPPV patients may be related to the simultaneous damage of multiple semicircular canals or bilateral vestibules in trauma.

The inferior treatment response in t-BPPV can be attributed to the severity of the initial insult. Blunt head trauma often causes diffuse damage to the utricular macula, leading to profuse otoconial debris. This explains why patients require significantly more maneuvers (often ≥2 sessions) to clear the semicircular canals. Clinically, these findings necessitate a paradigm shift: t-BPPV should not be managed identically to i-BPPV. We recommend extended follow-up periods and more aggressive repositioning protocols for trauma patients to mitigate the high recurrence rates observed. Contrary to absolute claims of zero bias, we acknowledge that heterogeneity was present (I^2^up to 59%). While Egger’s test indicated no publication bias, clinical heterogeneity arising from variations in trauma severity (mild concussion vs. severe TBI) and repositioning techniques is inevitable. Our strict inclusion of only high-quality cohort studies (NOS ≥ 6) and the use of random-effects models were employed specifically to mitigate these limitations.

Although we strictly follow the relevant standards and instructions in the analysis, there are still several limitations. ① The included outcome indicators are different, and the number of included studies of some outcome indicators is small, which affects the reliability of the conclusion; ② All the meta-analysis studies included in this meta-analysis were retrospective cohort studies, some data were lost to follow-up, and the heterogeneity of the included population in each literature was relatively large; ③ The type and intensity of trauma causing t-BPPV are different. Some are only mild traumatic brain injury, and some contain various types of TBI, such as moderate and severe TBI, resulting in obvious differences in the types and intensity of trauma in the included literatures, which leads to different clinical characteristics of t-BPPV and bias.

According to the review, this meta-analysis study shows that t-BPPV is significantly different from i-BPPV in five aspects, including recurrence rate, reduction difficulty, rehabilitation effect, proportion of the affected semicircular canals, proportion of bilateral BPPV. t-BPPV has poor reduction effect, is prone to residual symptoms after treatment, is prone to recurrence, and the incidence of bilateral BPPV is high. In view of the above characteristics of t-BPPV, we should be more patient and careful in clinical examination. After reduction, we should also instruct patients to return to the hospital in time if positional vertigo reappears. For those with frequent recurrence, they need drug treatment and long-term follow-up.

Future prospective, multicenter cohort studies with unified trauma grading and standardized follow-up time points are warranted to quantify the impact of different trauma types/severities on otolith organ injury. Integrating high-resolution inner-ear MRI with otolith proteomics will help elucidate the molecular–structural mechanisms underlying t-BPPV recurrence and residual symptoms. Randomized controlled trials comparing long-term efficacy of pharmacologically assisted repositioning, vestibular rehabilitation, and biomaterial-based repair for refractory t-BPPV are also needed to provide high-level evidence for individualized precision management.

## 5 Conclusion

The reduction effect of t-BPPV is poor, and it is easy to have residual symptoms and recurrence after treatment. The incidence of semicircular canals and bilateral BPPV is high.

## Data Availability

The original contributions presented in the study are included in the article/supplementary material, further inquiries can be directed to the corresponding author/s.
